# N^6^-Benzyladenosine Triggers β-Catenin Degradation to Induce Apoptotic and Ferroptotic Death in Colon Cancer Cells

**DOI:** 10.4014/jmb.2608.08023

**Published:** 2026-10-15

**Authors:** Yemin Han, Nahye Koo, Wonku Kang, Sangtaek Oh

**Affiliations:** 1 Department of Integrative Biotechnology, Kookmin University, Seoul 02707, Republic of Korea; 2 College of Pharmacy, Chung-Ang University, Seoul 06974, Republic of Korea

**Keywords:** N^6^-Benzyladenosine, Wnt/β-catenin signaling, Colon cancer, Apoptosis, Ferroptosis

## Abstract

N^6^-Benzyladenosine, an aromatic cytokinin, is cytotoxic toward diverse cancer cell types, yet how it prevents colon tumorigenesis and which molecular targets it engages have not been clearly established. Employing a reporter-based screening system, we show here that N^6^-Benzyladenosine antagonizes the Wnt/β-catenin cascade, a pathway commonly dysregulated in colorectal malignancy. Exposure to N^6^-Benzyladenosine lowered β-catenin-responsive transcription (CRT) by accelerating proteasome-mediated clearance of cytoplasmic β-catenin, and this effect persisted in colon cancer cell lines bearing β-catenin or adenomatous polyposis coli (APC) mutations. N^6^-Benzyladenosine further down-regulated cyclin D1 and c-Myc—two effectors controlled by the β-catenin/TCF complex—and curtailed colon cancer cell growth. Treatment additionally drove colon cancer cells toward apoptosis, evidenced by a rise in Annexin V-FITC-positive populations and enhanced caspase-3/7 activity, and toward ferroptosis, marked by diminished GPX4 protein, elevated labile Fe^2+^, and lipid peroxide accumulation. Together, these data indicate that N^6^-Benzyladenosine restrains colon cancer cell growth by depleting the pool of β-catenin that is unphosphorylated at Ser33/37/Thr41 and accelerating its proteasome-dependent turnover, pointing to its potential utility as a chemopreventive agent against colorectal cancer.

## Introduction

Cell growth, lineage commitment, and tissue patterning depend heavily on Wnt/β-catenin signaling [1]. Cytosolic β-catenin abundance is normally kept in check by a proteasome-dependent turnover mechanism operating within this pathway [2]. Signaling begins when secreted Wnt glycoproteins engage the Frizzled (Fz) receptor together with its LRP5/6 co-receptor [1]. In the unstimulated state, a multiprotein destruction complex—composed of adenomatous polyposis coli (APC), Axin, casein kinase 1 (CK1), and glycogen synthase kinase-3β (GSK-3β)—phosphorylates β-catenin at four N-terminal residues (Ser33, Ser37, Ser45, and Thr41), marking it for recognition by the F-box-containing E3 ubiquitin ligase β-TrCP and subsequent proteasomal destruction [1].

That phosphorylation is essential for β-catenin turnover is evident from the frequency with which these Ser/Thr sites are mutated across human cancers [3]. Colon cancer, in particular, is characterized by recurrent oncogenic alterations either directly in β-catenin or in destruction complex components such as Axin and APC. Such lesions allow β-catenin to accumulate in the cytosol and enter the nucleus, where it partners with TCF/LEF transcription factors to drive the expression of tumorpromoting and pro-metastatic genes, including *MYC*, *CCND1* (encoding cyclin D1), and *MMP7* (encoding matrix metalloproteinase-7) [3]. Given this central role, interventions that suppress Wnt/β-catenin signaling represent an attractive avenue for colon cancer chemoprevention and treatment [4].

Belonging to the aromatic cytokinin family of plant hormones, N^6^-Benzyladenosine also serves as one of the transport forms through which these phytohormones move within plants [5]. At micromolar concentrations, it curbs cell proliferation and triggers apoptosis, and a recent report describes its capacity to influence differentiation, growth, and programmed death in human myeloid leukemia cells [6]. Separately, ferroptosis has emerged as a form of regulated cell death that is mechanistically, morphologically, and genetically distinct from apoptosis, necrosis, and autophagy [7]. It arises from iron-catalyzed peroxidation of membrane polyunsaturated fatty acids, a reaction normally kept under control by the selenoprotein glutathione peroxidase 4 (GPX4), which converts damaging lipid hydroperoxides into harmless lipid alcohols [8]. When the GPX4 function is lost, and redox-active labile iron (Fe^2+^) together with reactive oxygen species accumulates, the resulting lipid peroxidation becomes lethal and drives ferroptotic death [9]. Colon cancer cells often depend on GPX4-based and related antioxidant systems to escape oxidative death, making pharmacological activation of ferroptosis an appealing chemopreventive and therapeutic approach.

Although the cytotoxic potential of N^6^-Benzyladenosine is well established, the mechanistic basis of its chemopreventive activity in colon cancer—and specifically whether it can engage cell-death programs such as ferroptosis—has not been examined. Here, we report that N6-Benzyladenosine is cytotoxic to colon cancer cells by inhibiting Wnt/β-catenin signaling via a route of β-catenin degradation that does not appear to require GSK-3β, coupled with concurrent activation of apoptotic and ferroptotic death.

## Materials and Methods

### Cell Cultures, Reporter Assays, and Chemicals

HEK293, SW480, HCT116 and Wnt3a-secreting L cells were sourced from the American Type Culture Collection (USA) and cultured in DMEM containing 10% fetal bovine serum (FBS) and 1% penicillin/streptomycin [10]. The HEK293-FL (TOPFlash) and HEK293-SEAP reporter lines were established as previously described [11, 12]. For the preparation of Wnt3a-conditioned medium (Wnt3a-CM), Wnt3a-secreting L cells were cultured for 4 days. The culture medium was collected and replaced with fresh medium, and the cells were cultured for an additional 3 days. The collected media were combined and filtered through a 0.22-μm membrane filter [11]. Luciferase and SEAP activities were measured using the Dual-Luciferase^®^ Reporter Assay System (Promega, Madison, WI, USA) and the Phospha-Light SEAP Reporter Gene Assay System (Applied Biosystems, Foster City, CA, USA), respectively, according to the manufacturers' instructions. Lithium chloride (LiCl), N^6^-Benzyladenosine, and MG-132 were purchased from Sigma-Aldrich (St. Louis, MO, USA).

### Western Blot Analysis

Cytosolic and nuclear protein fractions were isolated, resolved by SDS-PAGE, transferred, and immunoblotted as previously described [10, 13]. Primary antibodies used in this study included β-catenin (1:1000, BD Transduction Laboratories, USA), cyclin D1 (1:1000, ABclonal, China), c-Myc (1:1000, ABclonal, China), PARP (1:1000, Cell Signaling Technology, USA), non-phospho (active) β-catenin (Ser33/37/Thr41) (1:1000, Cell Signaling Technology, USA), cleaved caspase-3 (1:1000, Cell Signaling Technology, USA), GPX4 (1:1000, ABclonal, China), and actin (1:2000, Sigma-Aldrich, USA). After primary antibody incubation, membranes were rinsed three times with Tris-buffered saline containing 0.1% Tween-20 (TBS-T) for 10 min each at room temperature. Subsequently, horseradish peroxidase (HRP)-conjugated secondary antibodies were applied for 1 h at room temperature: anti-mouse IgG (1:1000, Santa Cruz Biotechnology, USA, sc-516102) or anti-rabbit IgG (1:1000, Santa Cruz Biotechnology, USA, sc-2357). The membranes were then washed again three times with TBS-T to remove unbound antibodies. The Chemidoc MP imaging system (Bio-Rad, USA) and Image J software (NIH, USA) were used to visualize and quantify protein bands.

### RNA Isolation and Semi-Quantitative RT-PCR

Total RNA isolation, cDNA synthesis, and PCR amplification were performed as previously described [10, 11]. TRIzol reagent (Invitrogen, USA) was used to isolate total RNA according to the manufacturer’s instructions. Amplified products were resolved on 2% agarose gels and visualized with Loading Star staining (Dynebio, Republic of Korea). Primer sequences were: β-catenin forward, 5′-GGGATGTTCACAA CCGAATTGTTATG-3′, and reverse, 5′-ACCAGAGTGAAAAGAACGATAGCTAGGA-3′; GAPDH forward, 5′-ACCACAGTCCATGCC ATCAC-3′, and reverse, 5′-TCCACCACCCTGTTGCTGTA-3′.

### Cell Viability Assay

Cell viability was assessed as previously described [10]. Briefly, SW480 and HCT116 cells (0.5 × 10^4^ cells/well) were seeded in black 96-well plates, grown overnight, treated with N^6^-Benzyladenosine for 48 h, and evaluated in duplicate with the CellTiter-Glo Assay Kit (Promega, Madison, WI, USA) according to the manufacturer's protocol.

### Apoptosis Analysis

Apoptosis was evaluated by Annexin V-FITC/propidium iodide (PI) staining and image cytometry as previously described [10], using the ApoScan Annexin V-FITC Apoptosis Detection Kit (BioBud, Republic of Korea) and a Cellometer Vision image cytometer (Nexcelom Bioscience, USA).

### Caspase-3/7 Assay

Caspase-3/7 activity was measured as previously described [10]. Briefly, SW480 and HCT116 cells were seeded into a 96-well black-bottom plate at 4000 cells/well. After 48 hours of N^6^-Benzyladenosine treatment, the caspase-3/7 reagent was added to each well, and the luminescence signal was measured with a Victor 3 microplate reader.

### Measurement of Ferrous Ion Levels

Intracellular Fe^2+^ levels were measured with the FerroOrange probe as previously described [10]. Briefly, SW480 and HCT116 cells (1.5 × 10^4^ cells/well) were treated with N^6^-Benzyladenosine for 48 h, incubated with 1 μM FerroOrange (Dojindo, Japan) for 30 min at 37°C, and imaged using a Zeiss Axiovert 200M fluorescence microscope (Carl Zeiss, Germany).

### Determination of Lipid Reactive Oxygen Species

Lipid peroxidation was assessed with the C11-BODIPY581/591 probe (Invitrogen, USA) as previously described [10], using the same seeding density and N^6^-Benzyladenosine treatment conditions as the ferrous ion assay.

### Statistical Analysis

Statistical comparisons between groups were performed using Student's t-test. All experiments were performed in triplicate. Data are presented as mean ± standard deviation (SD). A p-value < 0.05 was considered statistically significant, while *p* < 0.01 indicated a more stringent level of statistical significance.

## Results

### N^6^-Benzyladenosine Identified as a Wnt/β-Catenin Pathway Inhibitor

A screen for suppressors of β-catenin-responsive transcription (CRT) was carried out in HEK293 cells stably expressing a synthetic β-catenin/TCF-driven firefly luciferase reporter (TOPFlash) together with human Frizzled-1 (hFz-1). N^6^-Benzyladenosine dampened Wnt3a-CM-stimulated TOPFlash activity in a dose-dependent fashion (Fig. 1A and 1B), and a parallel concentration-dependent suppression of Wnt3a-induced SEAP output was observed in HEK293-SEAP cells carrying the corresponding β-catenin/TCF-dependent SEAP reporter and hFz-1 (Fig. 1C). Together, these observations point to N^6^-Benzyladenosine as a selective inhibitor of Wnt/β-catenin pathway activity.

### Proteasome-Dependent Degradation of β-Catenin Is Enhanced by N^6^-Benzyladenosine

Western blotting with an anti-β-catenin antibody was used to track the impact of N^6^-Benzyladenosine on intracellular β-catenin. Consistent with the CRT results, Wnt3a-CM-induced cytosolic β-catenin accumulation in HEK293-FL cells was diminished by N^6^-Benzyladenosine treatment (Fig. 2A), whereas semi-quantitative RT-PCR revealed no corresponding change in β-catenin transcript levels (Fig. 2B), indicating a post-transcriptional mode of action. Given that proteasomal degradation normally governs β-catenin turnover [14], we tested whether the proteasome inhibitor MG-132 could block the N^6^-Benzyladenosine-induced decline in β-catenin. MG-132 co-treatment fully restored β-catenin levels in HEK293-FL cells otherwise treated with N^6^-Benzyladenosine (Fig. 2C), indicating that pathway suppression occurs via enhanced proteasomal β-catenin clearance. Because phosphorylation of β-catenin at its N-terminal Ser33/37/45 and Thr41 residues normally licenses ubiquitination and proteasomal breakdown in the absence of Wnt signaling [15], we next asked whether N^6^-Benzyladenosine affects this modification. Treatment lowered the level of β-catenin detected with an antibody that recognizes the protein only when it is unphosphorylated at Ser33, Ser37, and Thr41 (non-phospho (active) β-catenin; Fig. 2D), indicating that the fraction of β-catenin left unmodified at these destruction-complex target residues declines, consistent with N^6^-Benzyladenosine promoting phosphorylation at these residues and the subsequent proteasome-mediated degradation of β-catenin, with consequent suppression of Wnt/β-catenin signaling. Because this readout reports the loss of the non-phosphorylated species rather than the direct detection of individual phosphorylated residues, and because β-catenin ubiquitination was not assayed in this study, the involvement of the ubiquitin-proteasome system is inferred from the MG-132 reversibility of the response rather than demonstrated directly.

### β-Catenin Downregulation by N^6^-Benzyladenosine Occurs When GSK-3β Is Inhibited

Because GSK-3β is the principal kinase driving Ser33/Ser37/Thr41 phosphorylation and consequent degradation of β-catenin within the Wnt pathway [3], we asked whether N^6^-Benzyladenosine acts through this kinase. LiCl, a GSK-3β inhibitor that boosts CRT activity, was combined with N^6^-Benzyladenosine treatment of HEK293-FL cells; CRT levels still fell in a dose-dependent manner despite GSK-3β inhibition (Fig. 3A). Western blotting confirmed that N^6^-Benzyladenosine reduced both total β-catenin (Fig. 3B) and the fraction unphosphorylated at Ser33/37/Thr41 (Fig. 3C) even when these had been elevated by LiCl. These observations are consistent with N^6^-Benzyladenosine promoting β-catenin degradation through a mechanism that does not require GSK-3β activity.

### N^6^-Benzyladenosine Lowers β-Catenin and Its Downstream Targets in Colon Cancer Cells

Given that Wnt/β-catenin pathway mutations are present in most colorectal cancer (CRC) cases [16], with APC alterations alone accounting for roughly 80% of colon tumors and consequent β-catenin stabilization [17], we asked whether N^6^-Benzyladenosine could still act in cells already harboring such mutations. Western blotting of SW480 (APC-mutant) and HCT116 (β-catenin-mutant) cells showed a reduction in β-catenin protein following N^6^-Benzyladenosine treatment in both lines (Fig. 4A and 4B). This decline was accompanied by pronounced suppression of the β-catenin/TCF target proteins cyclin D1 and c-Myc (Fig. 4C and 4D), indicating that these downstream effectors track with β-catenin abundance and that N^6^-Benzyladenosine effectively dampens Wnt/β-catenin output in colon cancer cells.

### N^6^-Benzyladenosine Drives Apoptotic Death of Colon Cancer Cells

Since prior work links loss of β-catenin/TCF activity to apoptotic induction in colon cancer cells [18], we examined whether the viability effects of N^6^-Benzyladenosine reflect this mechanism. N^6^-Benzyladenosine dose-dependently lowered the viability of SW480 and HCT116 cells while sparing non-transformed CCD-18Co cells (Fig. 5A). Annexin V-FITC/PI staining and cytometric analysis of treated colon cancer cells revealed a concentration-dependent rise in the apoptotic (double-positive) population—from 0.88% to 11.4% in SW480 cells and from 0.51% to 18.08% in HCT116 cells at 40 μM N^6^-Benzyladenosine (Fig. 5B). This was paralleled by increased caspase-3/7 activity (Fig. 5C) and by the emergence of cleaved, active caspase-3 together with its downstream substrate cleaved PARP, a hallmark apoptotic marker (Fig. 5D). These data collectively support apoptosis as a mechanism underlying the anti-proliferative action of N^6^-Benzyladenosine in colon cancer cells.

### N^6^-Benzyladenosine Also Triggers Ferroptotic Death in Colon Cancer Cells

The selective cytotoxicity of N^6^-Benzyladenosine toward colon cancer cells, together with its engagement of apoptotic markers, prompted us to ask whether it also activates ferroptosis, an iron-driven death pathway that colon cancer cells frequently escape via the antioxidant selenoprotein GPX4. GPX4 protein levels fell in a concentration-dependent manner in N^6^-Benzyladenosine-treated SW480 and HCT116 cells (Fig. 6A and 6B). As GPX4 loss permits redox-active iron and lipid peroxides to build up, we monitored intracellular Fe^2+^ with the FerroOrange probe and lipid peroxidation with C11-BODIPY 581/591. Both readouts rose in a dose-dependent fashion following N^6^-Benzyladenosine treatment—FerroOrange fluorescence indicating labile Fe^2+^ accumulation (Fig. 6C) and oxidized C11-BODIPY signal indicating elevated lipid peroxidation (Fig. 6D) in both cell lines. Together with its pro-apoptotic effects, these results show that N^6^-Benzyladenosine also induces ferroptosis in colon cancer cells by suppressing GPX4 and allowing iron and lipid peroxides to accumulate.

## Discussion

As members of the aromatic cytokinin family, N^6^-Benzyladenosine and its benzyl-substituted analogs are recognized for anti-proliferative and pro-apoptotic activity across a range of human cancer types. A recent study showed that this compound arrests the cell cycle and triggers apoptosis in T24 bladder carcinoma cells, suggesting broader anticancer applicability [19]. Colorectal cancer (CRC) remains the third most frequently diagnosed malignancy and the second most common cause of cancer death worldwide [20], yet the mechanistic basis for N^6^-Benzyladenosine's anti-proliferative activity in colon cancer had not been elucidated [21]. This study addresses this gap, showing that N^6^-Benzyladenosine promotes phosphorylation at the destruction-complex target residues Ser33/37/Thr41 and proteasome-dependent degradation of β-catenin through a mechanism consistent with GSK-3β independence, while simultaneously activating apoptosis and ferroptosis in colon cancer cells.

When Wnt signaling is off, β-catenin is normally routed for destruction by a complex containing the tumor suppressors APC and Axin, the kinases GSK-3β and CK1, protein phosphatase 2A (PP2A), and the ubiquitin ligase β-TrCP [22]; mutations affecting the N-terminal CK1/GSK-3β phospho-sites of β-catenin, or in APC/Axin themselves, stabilize the protein by blunting this degradation [23]. Several lines of evidence from the present study suggest that N^6^-Benzyladenosine can act largely independently of this canonical machinery. β-Catenin remained subject to degradation even in the presence of GSK-3β inhibition achieved pharmacologically with LiCl, indicating that the process does not strictly depend on this kinase. Degradation likewise proceeded in APC-mutant SW480 cells, suggesting that functional APC is dispensable. Finally, β-catenin levels also fell in HCT116 cells, which carry a Ser45 (CK1 site) mutation, consistent with CK1 activity also not being essential. Because each of these conclusions rests on pharmacological or mutational evidence rather than on genetic ablation of the individual kinases, they are best read as support for, rather than formal proof of, a destruction-complex-independent route.

An alternative route for β-catenin phosphorylation at Ser33/37/Thr41, independent of the destruction complex, has been attributed to CDK2/cyclin A and PKCα [24-26]. Notably, N^6^-Benzyladenosine depleted the pool of β-catenin unphosphorylated at Ser33/37/Thr41, implying increased occupancy of these sites, even in cells stimulated with Wnt3a-CM, a condition that normally suppresses GSK-3β-mediated phosphorylation. However, this readout indicates the loss of the non-phosphorylated species rather than directly detecting individual phosphorylated residues. Additionally, β-catenin ubiquitination was not measured in this study; hence, the role of the ubiquitin-proteasome system is inferred from the MG-132 reversibility of the response, not confirmed directly. Moreover, because this study did not directly examine either kinase, CDK2 and PKCα should be regarded as hypotheses for future investigation.

Constitutive Wnt pathway activation underlies tumor initiation in an estimated 80–90% of colon cancers [27], and experimentally, Wnt3a-CM or LiCl exposure substantially boosts colon cancer cell proliferation [28], underscoring why blocking this pathway is a valued chemopreventive strategy. Precedents for this approach include USP44, which deubiquitinates Axin1 to inactivate Wnt/β-catenin signaling and thereby restrain proliferation while favoring apoptosis in colorectal cancer cells [29], and the opposing example of miR-15b, which promotes colon cancer growth and spread by elevating β-catenin through Axin2 suppression [30]. To our knowledge, this is the first report identifying N^6^-Benzyladenosine as an agent that curbs colon cancer cell proliferation by reducing intracellular β-catenin. The novelty of the present study therefore lies in two points. First, it extends the previously described anti-proliferative and pro-apoptotic activity of this aromatic cytokinin by identifying β-catenin stability as a molecular target of the compound. Second, it shows that apoptotic and ferroptotic death programs are engaged concurrently in the same colon cancer cells—a combination that, to our knowledge, has not previously been reported for this compound or for related cytokinin ribosides.

A number of natural products have been shown to restrain colon cancer cell growth via Wnt/β-catenin suppression, through mechanistically diverse routes: apigenin blocks proliferation, migration, and invasion by inhibiting the pathway directly [31]; the isoquinoline alkaloid berberine promotes c-Cbl-dependent β-catenin degradation by strengthening RXRα's interaction with nuclear β-catenin [32]; tetrandrine, a bis-benzylisoquinoline alkaloid, acts partly by lowering IGFBP-5 to inactivate Wnt/β-catenin signaling [33]; and toosendanin, derived from Melia toosendan bark and fruit, suppresses the AKT/GSK-3β/β-catenin axis to inhibit proliferation and induce apoptosis [34]. Our observation that MG-132 completely blocked N^6^-Benzyladenosine-induced β-catenin loss fits this broader pattern and indicates that N^6^-Benzyladenosine acts through proteasome-dependent β-catenin degradation to restrain Wnt/β-catenin signaling.

Beyond its pro-apoptotic activity, N^6^-Benzyladenosine lowered GPX4 and raised intracellular Fe^2+^ and lipid peroxide levels, marking ferroptosis as a second death pathway it engages in colon cancer cells [7-9]. As the principal brake on ferroptosis, GPX4 suppression alone is sufficient to sensitize many cancer cell types, colon cancer included, to iron-dependent oxidative injury [8]. Given documented links between Wnt/β-catenin signaling and genes governing redox and iron homeostasis, N^6^-Benzyladenosine-driven β-catenin loss may simultaneously silence proliferative/survival programs and erode the antioxidant defenses that would otherwise shield colon cancer cells from ferroptosis. Whether the apoptotic and ferroptotic outcomes of N^6^-Benzyladenosine treatment are causally tied to Wnt/β-catenin inhibition, or instead arise as independent, parallel effects, remains to be clarified in future work.

A key question is whether the 10–40 μM range used here is achievable *in vivo*. The potencies we observed align with those reported for aromatic and isoprenoid cytokinin ribosides, which typically have IC_50_ values between 0.5 and 50 μM against human cancer cell lines [35]. Similar ribosides have shown activity in xenograft models: kinetin riboside induced tumor regression in human myeloma xenografts when administered frequently to counteract its short plasma half-life [36], and N^6^-isopentenyladenosine reduced tumor weight by about 80% at 0.5 mg/kg per dose without evident toxicity [37]. Conversely, a systematic study of N^6^-isopentenyladenosine and related analogs found no significant *in vivo* activity, largely due to rapid metabolism [38]. While low-micromolar levels are theoretically achievable, rapid clearance and degradation by adenosine deaminase imply that dosing schedules, formulations, or metabolically stabilized analogs are crucial. Since no pharmacokinetic data are currently available for N^6^-Benzyladenosine, these results should be viewed as a mechanistic basis for further research into plasma and tumor exposure, therapeutic window, and xenograft efficacy, rather than concrete evidence of clinical potential.

Taken together, this work provides the first mechanistic account of how N^6^-Benzyladenosine restrains colon cancer cell proliferation: by promoting phosphorylation at Ser33/37/Thr41 and proteasome-dependent degradation of oncogenic β-catenin, through a mechanism consistent with GSK-3β independence, thereby silencing the β-catenin/TCF target genes *MYC* and *CCND1* and driving both apoptotic and ferroptotic death. Because the Wnt/β-catenin pathway occupies a central position in colon tumorigenesis, N^6^-Benzyladenosine warrants further consideration as a candidate chemopreventive or therapeutic agent for cancers carrying β-catenin or APC mutations.

## Figures and Tables

**Fig. 1:**
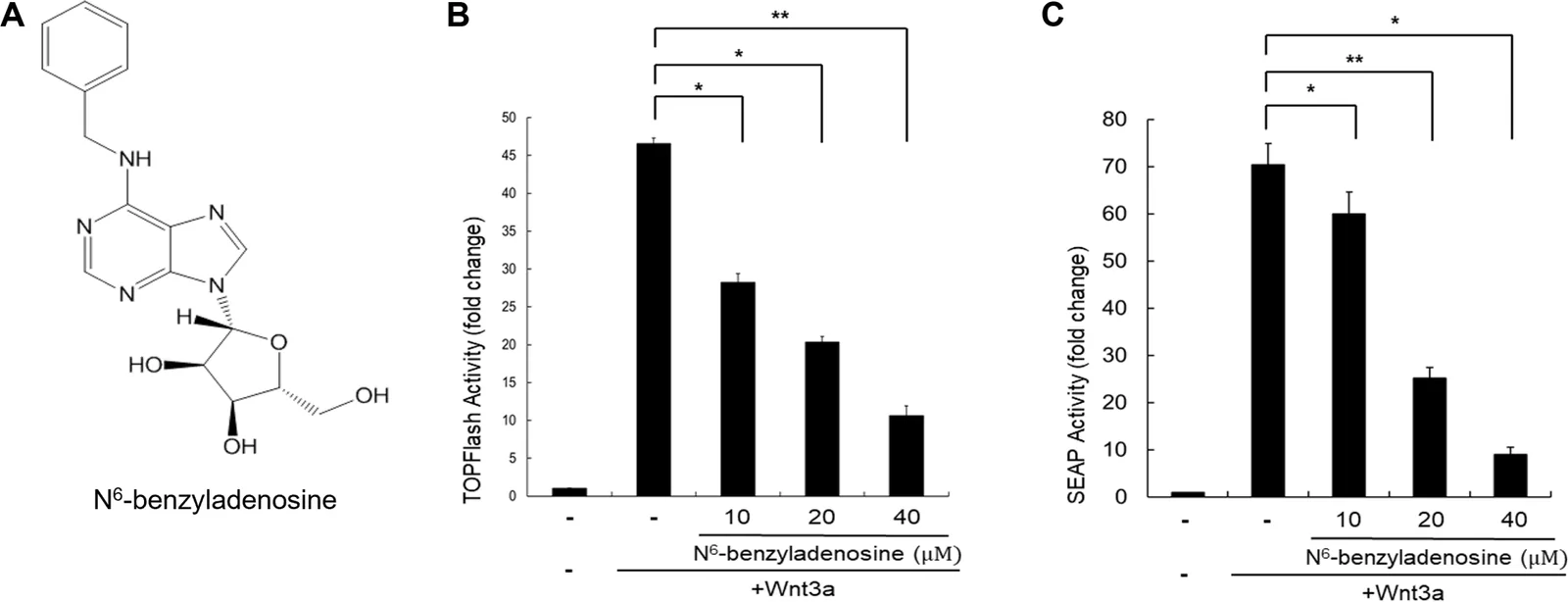
N^6^-Benzyladenosine antagonizes Wnt/β-catenin pathway activity. (**A**) Structure of the N^6^-Benzyladenosine molecule. (**B, C**) HEK293-FL and HEK293-SEAP reporter cells received DMSO or the indicated N^6^-Benzyladenosine concentrations together with Wnt3a-CM; both reporters show dose-dependent suppression. Data are mean ± SD (n = 3 independent experiments); *p < 0.05, **p < 0.01 vs. the Wnt3a-CM-only control group.

**Fig. 2:**
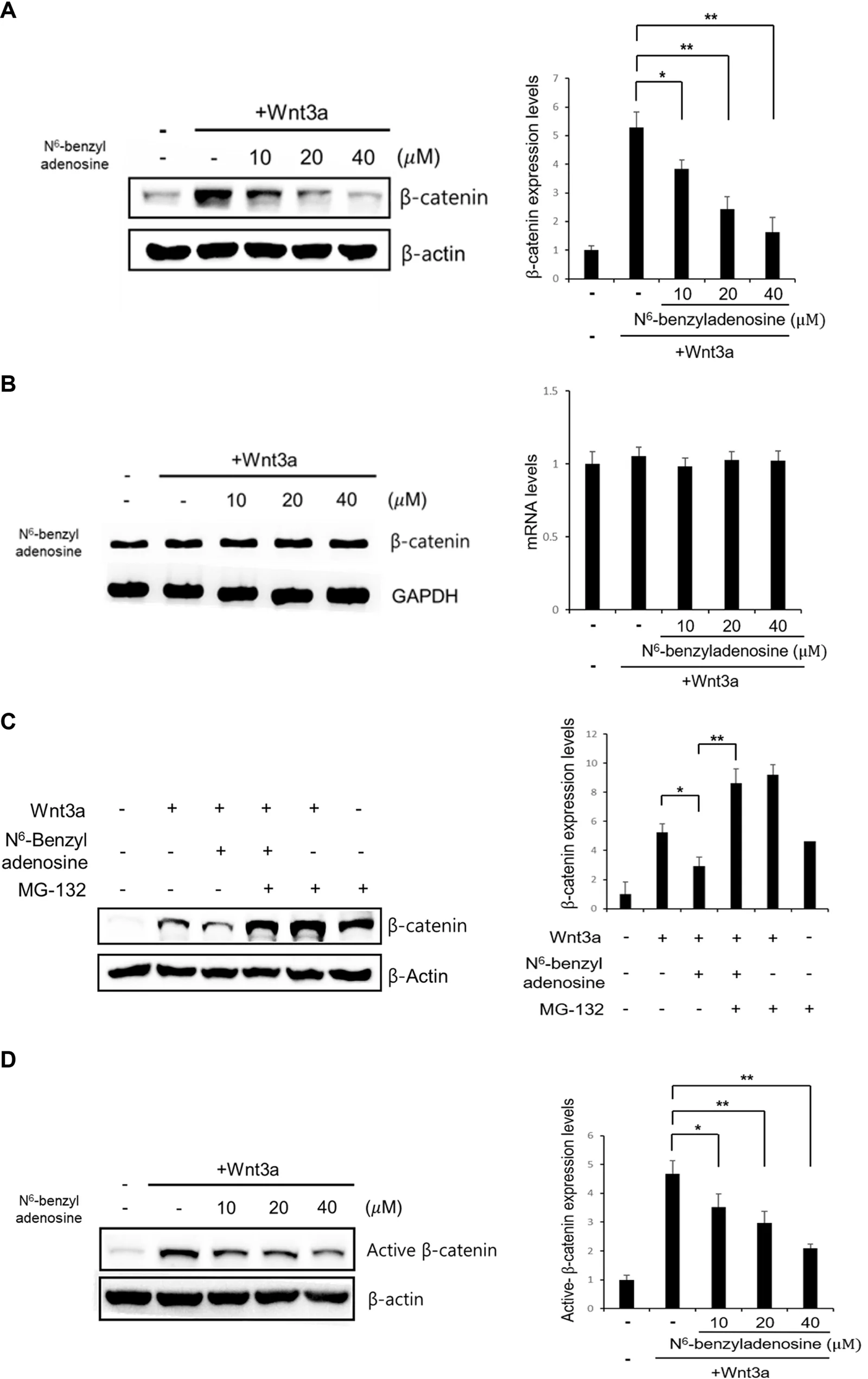
N^6^-Benzyladenosine drives proteasome-dependent β-catenin degradation. Bars show band density normalized to β-actin (**A, C, D**) or GAPDH (**B**) (mean ± SD, n = 3). Statistical significance was determined using Student's t-test; *p < 0.05, **p < 0.01 for the comparisons indicated by brackets.

**Fig. 3:**
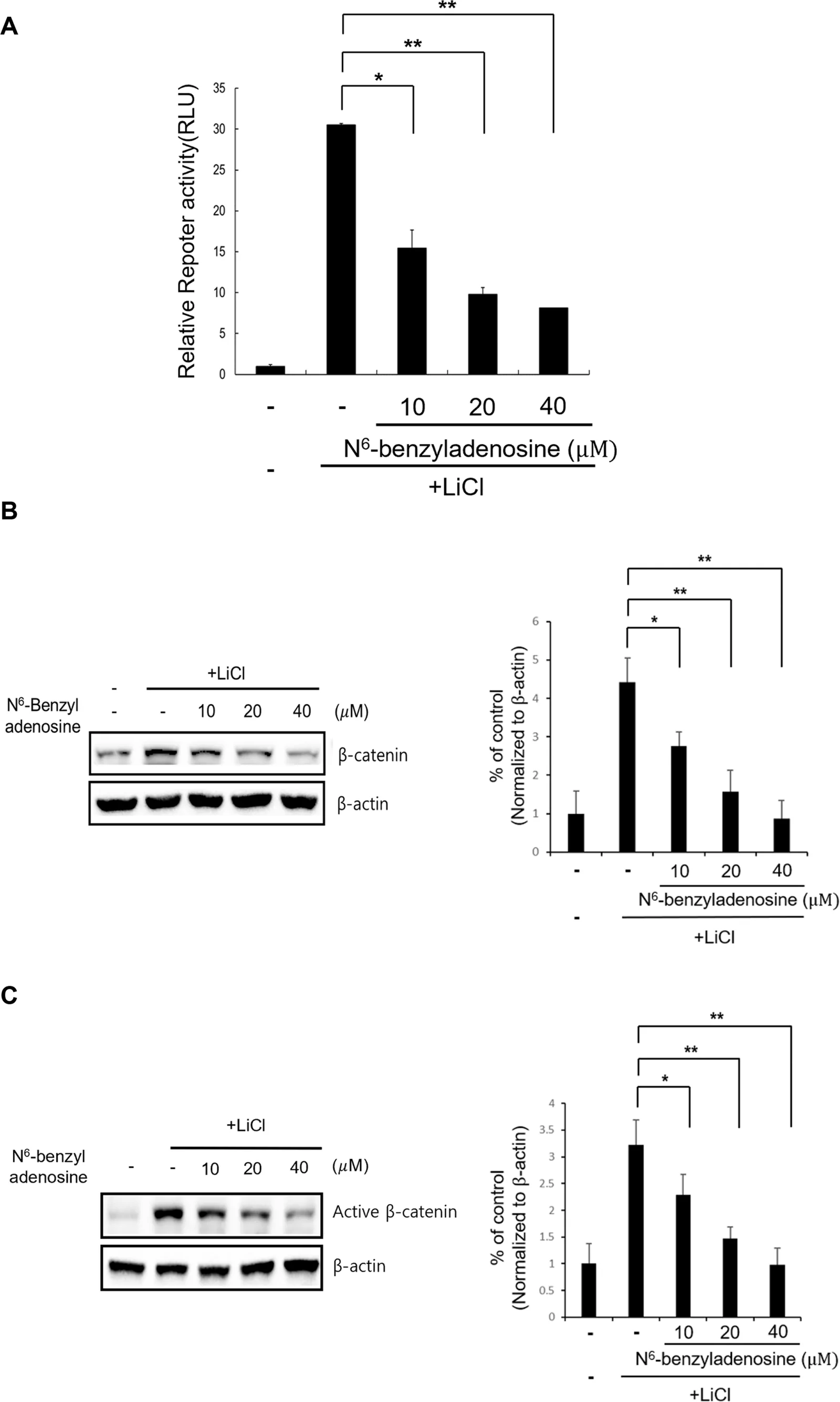
N^6^-Benzyladenosine reduces β-catenin levels under conditions of GSK-3β inhibition. (**A**) HEK293-FL reporter cells were treated with the indicated N^6^-Benzyladenosine concentrations in the presence of 20 mM LiCl, and FL activity was measured after 15 h. Data are mean ± SD of three independent experiments; *p < 0.05, **p < 0.01 vs. the LiCl-treated control. (**B, C**) HEK293-FL cells were treated with DMSO or N^6^-Benzyladenosine (10, 20, or 40 μM) plus 20 mM LiCl for 15 h; cytosolic β-catenin (**B**) and non-phospho (active) β-catenin (Ser33/37/Thr41) (**C**) were detected by western blotting, with β-actin as loading control. Bars show band density normalized to β-actin (mean ± SD, n = 3); *p < 0.05, **p < 0.01 vs. the LiCl-treated control.

**Fig. 4:**
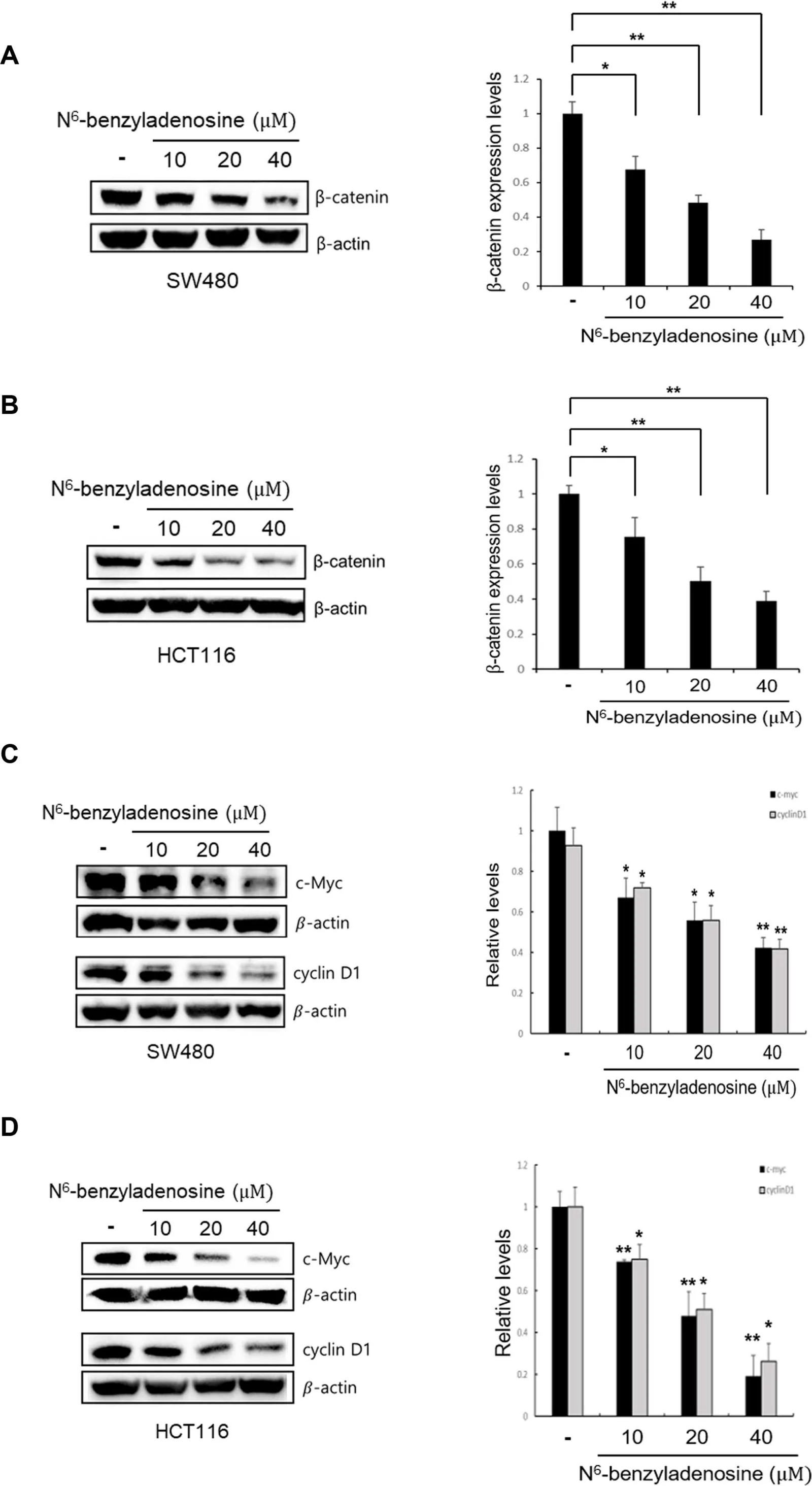
N^6^-Benzyladenosine reduces β-catenin and its downstream targets in colon cancer cells. (**A, B**) SW480 and HCT116 cells were treated with N^6^-Benzyladenosine (10, 20, or 40 μM) for 15 h, and cytosolic β-catenin was analyzed by western blotting. (**C, D**) The same cell lines were treated with N^6^-Benzyladenosine (10, 20, or 40 μM) for 48 h, and whole-cell extracts were probed for cyclin D1 and c-Myc. β-Actin served as the loading control throughout. Bars show band density normalized to β-actin (mean ± SD, n = 3); *p < 0.05, **p < 0.01 vs. the DMSO control.

**Fig. 5:**
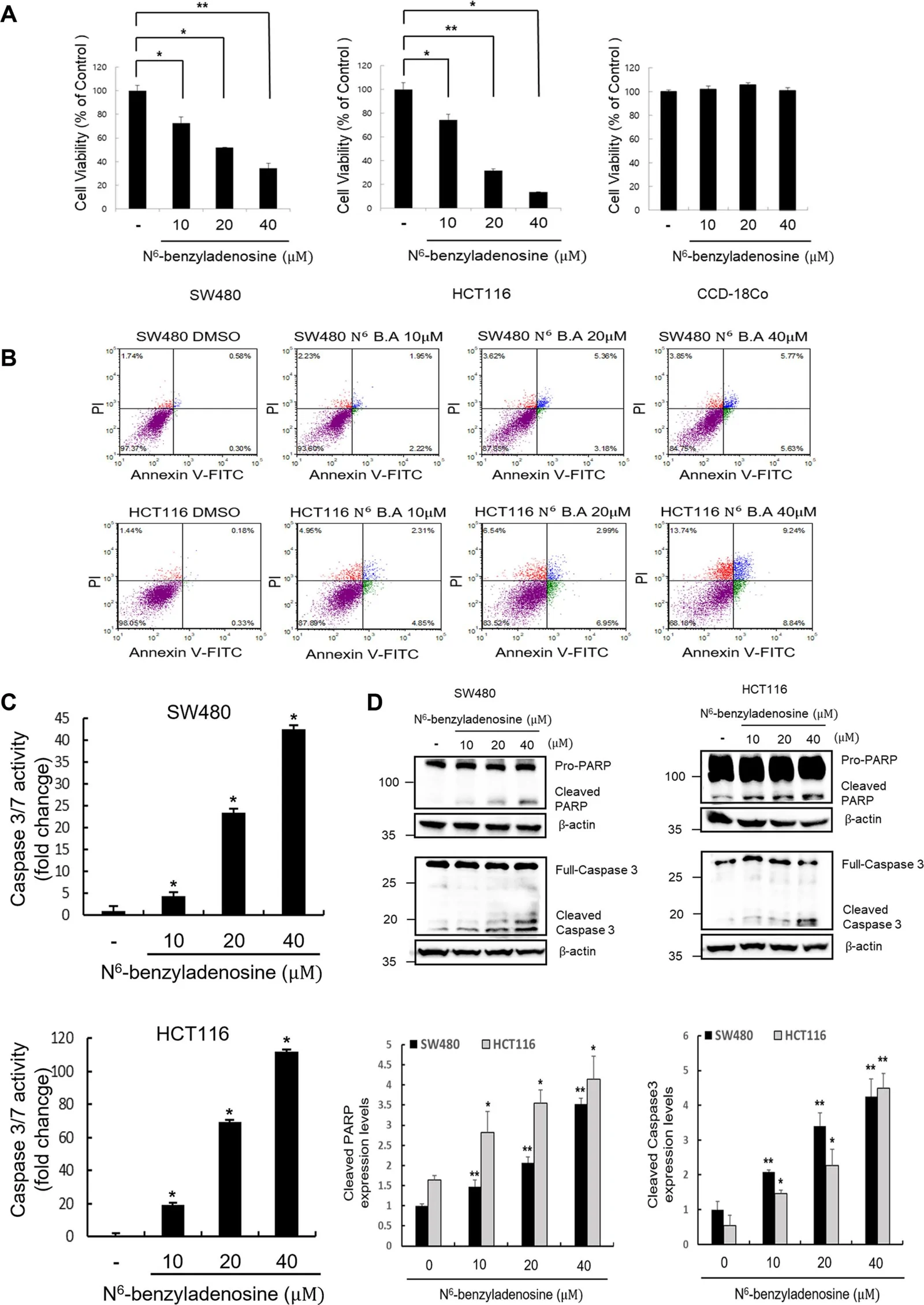
N^6^-Benzyladenosine induces apoptotic death of colon cancer cells. (**A**) SW480, HCT116, and CCD-18Co cell viability after 48-h N^6^-Benzyladenosine treatment (10, 20, or 40 μM), measured by CellTiter-Glo. (**B**) SW480 and HCT116 cells treated with DMSO or N^6^-Benzyladenosine (10, 20, or 40 μM, 48 h) were stained with Annexin V-FITC/PI; axes show Annexin V-FITC and PI fluorescence. (**C**) Caspase-3/7 activity in colon cancer cells after 48-h N^6^-Benzyladenosine treatment (mean ± SD, n = 3); *p < 0.05, **p < 0.01 vs. vehicle control. (**D**) Cell extracts from N^6^-Benzyladenosine-treated colon cancer cells were probed for cleaved caspase-3 and PARP, with β-actin as loading control. Bars show band density normalized to β-actin (mean ± SD, n = 3); *p < 0.05, **p < 0.01 vs. vehicle control.

**Fig. 6:**
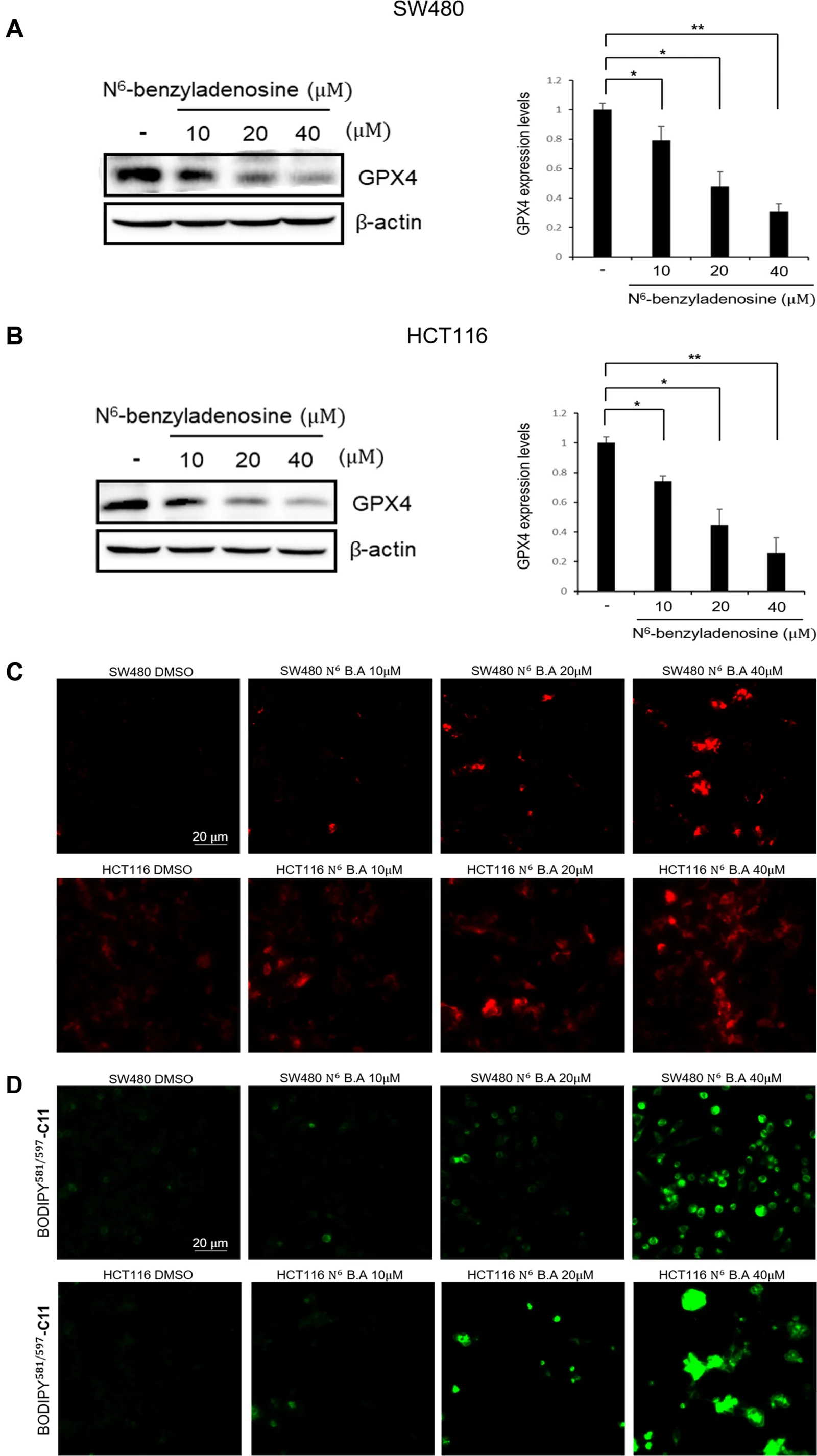
N^6^-Benzyladenosine induces ferroptosis in colon cancer cells. (**A, B**) SW480 and HCT116 cells treated with DMSO or N^6^-Benzyladenosine (48 h) were probed for GPX4 by western blotting, with β-actin as loading control. Bars show band density normalized to β-actin (mean ± SD, n = 3); *p < 0.05, **p < 0.01 vs. vehicle control. (**C**) Fluorescence images of FerroOrange-labeled intracellular Fe^2+^ in N^6^-Benzyladenosine-treated SW480 and HCT116 cells; intensity was quantified with ImageJ. (**D**) Fluorescence images of C11-BODIPY 581/591-labeled lipid peroxidation in N^6^-Benzyladenosine-treated SW480 and HCT116 cells; intensity was quantified with ImageJ.

## References

[1] Giles RH, Van Es JH, Clevers H (2003). Caught up in a Wnt storm: Wnt signaling in cancer. Biochim. Biophys. Acta.

[2] Hart MJ, de los Santos R, Albert IN, Rubinfeld B, Polakis P (1998). Downregulation of β-catenin by human Axin and its association with the APC tumor suppressor, β-catenin and GSK3β. Curr. Biol..

[3] Liu C, Li Y, Semenov M, Han C, Baeg GH, Tan Y (2002). Control of β-catenin phosphorylation/degradation by a dual-kinase mechanism. Cell.

[4] He TC, Sparks AB, Rago C, Hermeking H, Zawel L, da Costa LT (1998). Identification of c-MYC as a target of the APC pathway. Science.

[5] Štarha P, Popa I, Trávníček Z, Vančo J (2013). N^6^-Benzyladenosine derivatives as novel N-donor ligands of platinum(II) dichlorido complexes. Molecules.

[6] Ishii Y, Hori Y, Sakai S, Honma Y (2002). Control of differentiation and apoptosis of human myeloid leukemia cells by cytokinins and cytokinin nucleosides, plant redifferentiation-inducing hormones. Cell Growth Differ..

[7] Dixon SJ, Lemberg KM, Lamprecht MR, Skouta R, Zaitsev EM, Gleason CE (2012). Ferroptosis: an iron-dependent form of nonapoptotic cell death. Cell.

[8] Yang WS, SriRamaratnam R, Welsch ME, Shimada K, Skouta R, Viswanathan VS (2014). Regulation of ferroptotic cancer cell death by GPX4. Cell.

[9] Yang WS, Stockwell BR (2016). Ferroptosis: death by lipid peroxidation. Trends Cell Biol..

[10] Kim S, Na M, Oh S (2025). 1,2,3,6-Tetra-O-Galloyl-β-D-Glucopyranose Induces Apoptosis and Ferroptosis in Colon Cancer Cells by Inhibiting the Wnt/β-Catenin Signaling Pathway. J. Microbiol. Biotechnol..

[11] Park S, Gwak J, Han SJ, Oh S (2006). Hexachlorophene inhibits Wnt/β-catenin pathway by promoting Siah-mediated β-catenin degradation. Mol. Pharmacol..

[12] Ryu M-J, Cho M, Song J-Y, Yun Y-S, Choi I-W, Kim D-E (2008). Natural derivatives of curcumin attenuate the Wnt/β-catenin pathway through down-regulation of the transcriptional coactivator p300. Biochem. Biophys. Res. Commun..

[13] Dignam JD, Lebovitz RM, Roeder RG (1983). Accurate transcription initiation by RNA polymerase II in a soluble extract from isolated mammalian nuclei. Nucleic Acids Res..

[14] Aberle H, Bauer A, Stappert J, Kispert A, Kemler R (1997). β-catenin is a target for the ubiquitin-proteasome pathway. EMBO J..

[15] Hagen T, Vidal-Puig A (2002). Characterisation of the phosphorylation of β-catenin at the GSK-3 priming site Ser45. Biochem. Biophys. Res. Commun..

[16] Sebio A, Kahn M, Lenz HJ (2014). The potential of targeting Wnt/β-catenin in colon cancer. Expert Opin. Ther. Targets.

[17] Morin PJ (1999). β-catenin signaling and cancer. Bioessays.

[18] D. Xiang, Wang D, He Y, Xie J, Zhong Z, Li Z, et al. 2006. Caffeic acid phenethyl ester induces growth arrest and apoptosis of colon cancer cells via the β-catenin/T-cell factor signaling. *Anticancer Drugs* **17:** 753-762. http://doi.org/10.1097/01.cad.0000224441.01082.bb. 10.1097/01.cad.0000224441.01082.bb 16926625

[19] Castiglioni S, Casati S, Ottria R, Ciuffreda P, Maier JA (2013). N6-isopentenyladenosine and its analogue N^6^-benzyladenosine induce cell cycle arrest and apoptosis in bladder carcinoma T24 cells. Anticancer Agents Med. Chem..

[20] Malki A, ElRuz RA, Gupta I, Allouch A, Vranic S, Al Moustafa AE (2020). Molecular mechanisms of colon cancer progression and metastasis: recent insights and advancements. Int. J. Mol. Sci..

[21] de Sousa EMF, Vermeulen L, Richel D, Medema JP (2011). Targeting Wnt signaling in colon cancer stem cells. Clin. Cancer Res..

[22] Stamos JL, Weis WI (2013). The β-catenin destruction complex. Cold Spring Harb. Perspect. Biol..

[23] Barker N, Clevers H (2000). Catenins, Wnt signaling and cancer. Bioessays.

[24] Park CS, Kim SI, Lee MS, Youn C-Y, Kim DJ, Jho E-H (2004). Modulation of β-catenin phosphorylation/degradation by cyclin-dependent kinase 2. J. Biol. Chem..

[25] Gwak J, Cho M, Gong S-J, Won J, Kim D-E, Kim E-Y (2006). Protein-kinase-C-mediated β-catenin phosphorylation negatively regulates the Wnt/β-catenin pathway. J. Cell Sci..

[26] Gwak J, Jung S-J, Kang D-I, Kim E-Y, Kim D-E, Chung Y-H (2009). Stimulation of protein kinase C-α suppresses colon cancer cell proliferation by down-regulation of β-catenin. J. Cell. Mol. Med..

[27] Kinzler KW, Vogelstein B (1996). Lessons from hereditary colorectal cancer. Cell.

[28] Shin S, Son Y, Liu KH, Kang W, Oh S (2019). Cytotoxic activity of broussochalcone A against colon and liver cancer cells by promoting destruction complex-independent β-catenin degradation. Food Chem. Toxicol..

[29] Huang T, Zhang Q, Ren W, Yan B, Yi L, Tang T (2020). USP44 suppresses proliferation and enhances apoptosis in colorectal cancer cells by inactivating the Wnt/β-catenin pathway via Axin1 deubiquitination. Cell Biol. Int..

[30] Guo X, Zhang Y, Li J, Liao M (2016). miR-15b facilitates the progression of colorectal cancer via targeting β-catenin and Axin2. Int. J. Clin. Exp. Pathol..

[31] Xu M, Wang S, Song YU, Yao J, Huang K, Zhu X (2016). Apigenin suppresses colorectal cancer cell proliferation, migration and invasion via inhibition of the Wnt/β-catenin signaling pathway. Oncol. Lett..

[32] Ruan H, Zhan Y, Hou J, Xu B, Chen B, Tian Y (2017). Berberine binds RXRα to suppress β-catenin signaling in colon cancer cells. Oncogene.

[33] Wu K, Zhou M, Wu QX, Yuan SX, Wang DX, Jin JL (2015). The role of IGFBP-5 in mediating the anti-proliferation effect of tetrandrine in human colon cancer cells. Int. J. Oncol..

[34] Wang G, Feng C-C, Chu S-J, Zhang R, Lu Y-M, Zhu J-S (2015). Toosendanin inhibits growth and induces apoptosis in colorectal cancer cells through suppression of the AKT/GSK-3β/β-catenin pathway. Int. J. Oncol..

[35] Voller J, Zatloukal M, Lenobel R, Doležal K, Béreš T, Kryštof V (2010). Anticancer activity of natural cytokinins: a structure-activity relationship study. Phytochemistry.

[36] Tiedemann RE, Mao X, Shi CX, Zhu YX, Palmer SE, Sebag M (2008). Identification of kinetin riboside as a repressor of *CCND1* and *CCND2* with preclinical antimyeloma activity. J. Clin. Invest..

[37] Laezza C, Notarnicola M, Caruso MG, Messa C, Macchia M, Bertini S (2006). N^6^-isopentenyladenosine arrests tumor cell proliferation by inhibiting farnesyl diphosphate synthase and protein prenylation. FASEB J..

[38] Colombo F, Falvella FS, Tortoreto M, Pratesi G, Ciuffreda P, Ottria R (2009). Pharmacogenomics and analogues of the antitumour agent N^6^-isopentenyladenosine. Int. J. Cancer.

